# Treatment of central giant cell lesions using bisphosphonates with intralesional corticosteroid injections

**DOI:** 10.1186/1746-160X-8-23

**Published:** 2012-08-22

**Authors:** Newton Guerreiro da Silva, Aline Semblano Dias Carreira, Erick Nelo Pedreira, Fabrício Mesquita Tuji, Karem López Ortega, João de Jesus Viana Pinheiro

**Affiliations:** 1 School of Dentistry, Federal University of Pará-UFPA, Avenida Augusto Corrêa, 01, Belém, PA, 66075-110, Brazil; 2 Department of Oral and Maxillofacial Pathology, School of Dentistry, Federal University of Pará-UFPA, Rua dos Mundurucus, 4487, Belém, PA, 66073-000, Brazil; 3 School of Dentistry, University of São Paulo-USP, Avenida Professor Lineu Prestes, 2227, São Paulo, SP, 05508-000, Brazil; 4 Faculdade de Odontologia, Instituto de Ciências da Saúde, Universidade Federal do Pará, Avenida Augusto Corrêa, 01, Belém, PA, 66075-110, Brazil

**Keywords:** Giant central granuloma, Nonsurgical management, Corticosteroids, Bisphosphonates

## Abstract

Central giant cell lesions are benign intraosseous proliferative lesions that have considerable local aggressiveness. Nonsurgical treatment methods, such as intralesional corticosteroid injections, systemic calcitonin and interferon have been reported. Recently, bisphosphonates have been used to treat central giant cell lesions. A case of a 36-year-old male with a central giant cell lesion crossing the mandibular midline was treated with intralesional corticosteroids combined with alendronate sodium for the control of systemic bone resorption. The steroid injections and the use of bisphosphonates were stopped after seven months when further needle penetration into the lesion was not possible due to new bone formation. After two years, the bony architecture was near normal, and only minimal radiolucency was present around the root apices of the involved teeth. The patient was followed up for four years, and panoramic radiography showed areas of new bone formation. Thus far, neither recurrence nor side effects of the medication have been detected.

## Background

Central giant cell lesions (CGCLs) are benign intraosseous proliferative lesions that occur in the maxilla and mandible primarily during the first to third decades of life [1]. Histologically, multinucleated giant cells are prominent throughout the fibroblastic stroma and are often clustered around areas of haemorrhage [2].

CGCLs represent a treatment challenge. The clinical behaviour is extremely variable in that certain lesions are completely silent and grow very slowly whereas others behave more aggressively [3]. In recurrent or aggressive lesions, en bloc resection is a treatment option, but this procedure results in large surgical defects [4,5]. Nonsurgical treatment methods, such as intralesional corticosteroid injections and systemic calcitonin or interferon-α, are increasingly being used [4,6-11].

Steroids appear to inhibit the production of extracellular lysosomal proteases in multinucleated giant cells. In addition, steroids reduce bone resorption and induce the apoptosis of osteoclastic cells [5].

Bisphosphonates are widely used to inhibit osteolysis in conditions such as osteoporosis, Paget’s disease and bone destruction through metastatic cancer. Landesberg et al. [1] reported three cases of CGCLs treated with bisphosphonates. The first case showed the successful treatment with a single administration of intravenous bisphosphonates, the second resulted in a 30% reduction of the lesion, and the last case showed stabilisation but no regression of the lesion.

Based on such evidence, the purpose of this study was to report a case of CGCL that had been treated with intralesional corticosteroids and oral bisphosphonates.

## Report of a case

A 36-year-old male with no relevant medical history was referred for the evaluation of swelling on the left side of his jaw, which had been associated with paraesthesia for the previous 5 months. On extra-oral examination, a slight swelling in the chin region was noted. Intraorally, the examination revealed swelling of the mouth vestibule and high tooth mobility.

**Figure 1:**
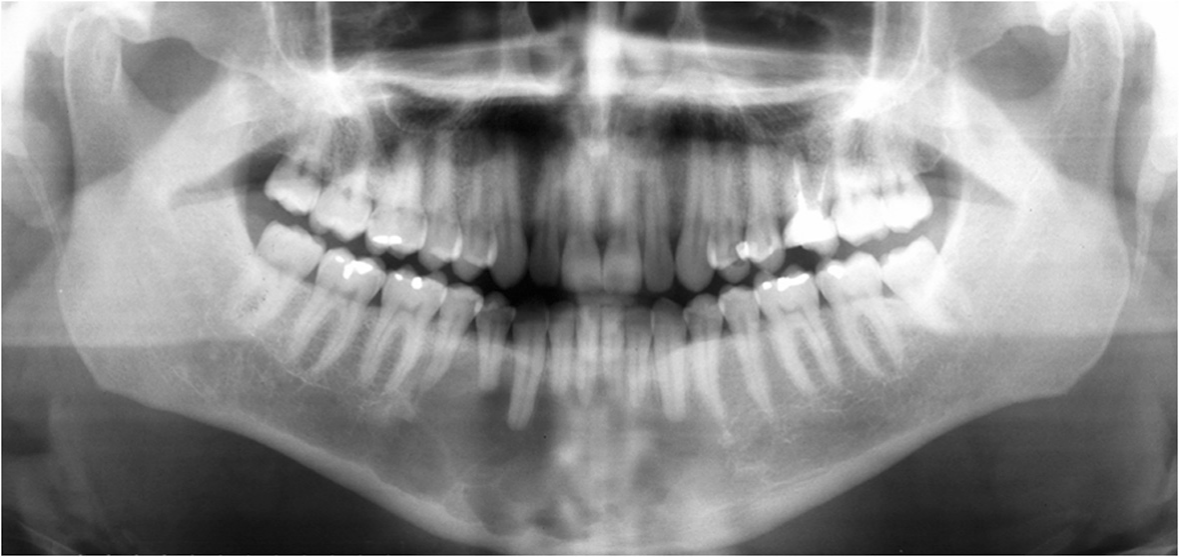
**A Panoramic radiography.** A multilocular radiolucent area crossing the midline and causing resorption in certain teeth.

**Figure 2:**
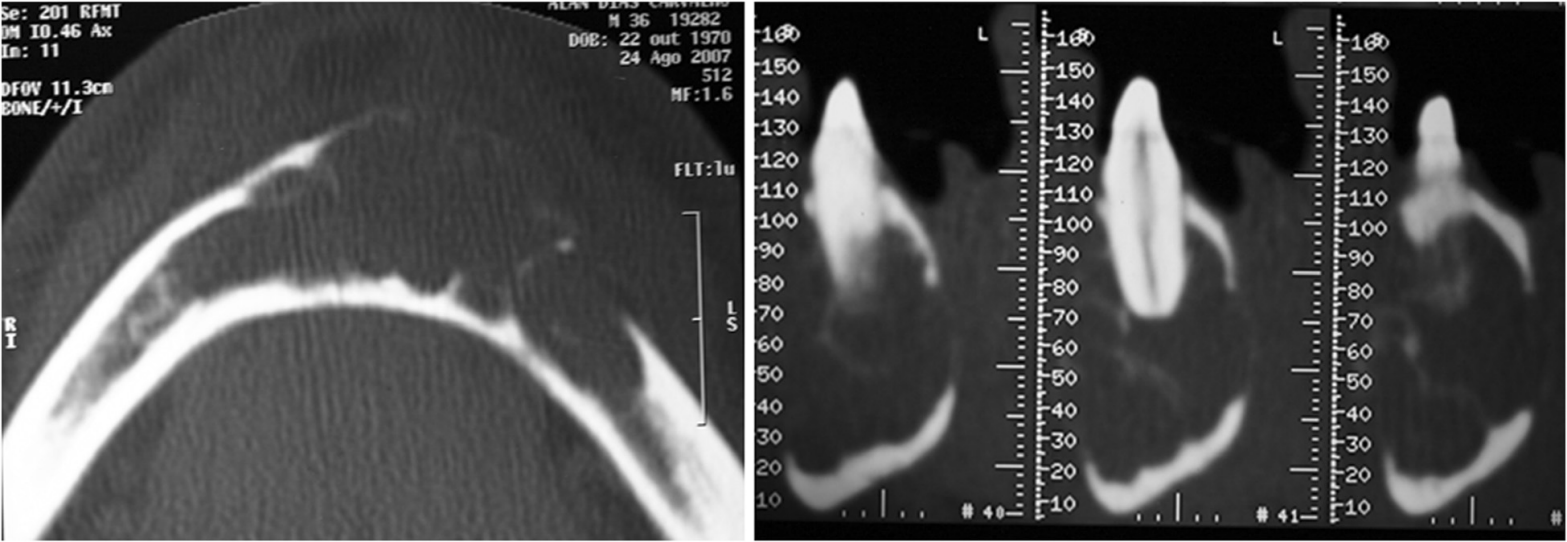
**Computed tompography scan.** The expansion of the buccal and lingual cortices with consequent disruption.

Panoramic radiographs showed a multilocular radiolucent area in the anterior mandible extending from tooth 36 to tooth 45, causing the resorption of certain teeth (Figure 1). A computed tomography (CT) scan showed that the lesion crossed the midline and extended to the inferior border of the mandible. In addition, there was an expansion of the buccal and lingual cortices with consequent disruption. The mandibular canal on both sides and the mandibular foramen on the left side were compromised (Figure 2).

**Figure 3:**
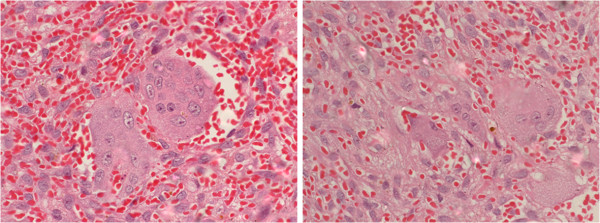
**Histological sections stained with H.E.** The presence of multinucleated giant cells surrounded by a disorganised stroma with an intense inflammatory infiltrate.

An incisional biopsy was performed, which showed multinucleated giant cells surrounded by a disorganised stroma with intense inflammatory infiltrates. There were mainly mononuclear infiltrates with numerous haemorrhagic areas and viable bone tissue surrounding the lesion. Based on these characteristics, the final diagnosis was CGCL (Figure 3).

The results of blood tests were normal, and after ruling out the possibility of the injury being associated with hormonal disorders (hyperparathyroidism), the proposed treatment was intralesional infiltration of triamcinolone, similar to the protocol described by Terry and Jacoway [12]. Such infiltrations are typically recommended to be performed weekly for six weeks at multiple sites of the lesion [5,6,9].

In this case, a 1 ml ampoule of Theracort (triamcinolone acetonide, 40 mg/ml) diluted in 3,6 ml of 2% mepivacaine (epinephrine 1:100,000) was used. The patient received 10 injections, seven of which were given over a period of approximately 15 days and the remaining three injections over the course of one month. The corticosteroid solution was more concentrated in a specific area of the lesion in each infiltration. After the first injection, the anaesthetic solution was replaced by 3% mepivacaine (without epinephrine) because the patient was tachycardic during infiltration even when the procedure was performed slowly for approximately five minutes.

Alendronate sodium (70 mg) was used on a weekly basis to control bone resorption during treatment. Additionally, oral calcium carbonate (500 mg) was administered daily to facilitate the calcification of the lesion. The treatment lasted seven months, after which further needle penetration into the lesion was not possible due to new bone formation. The administration of alendronate sodium and calcium carbonate was suspended at the same time.

**Figure 4:**
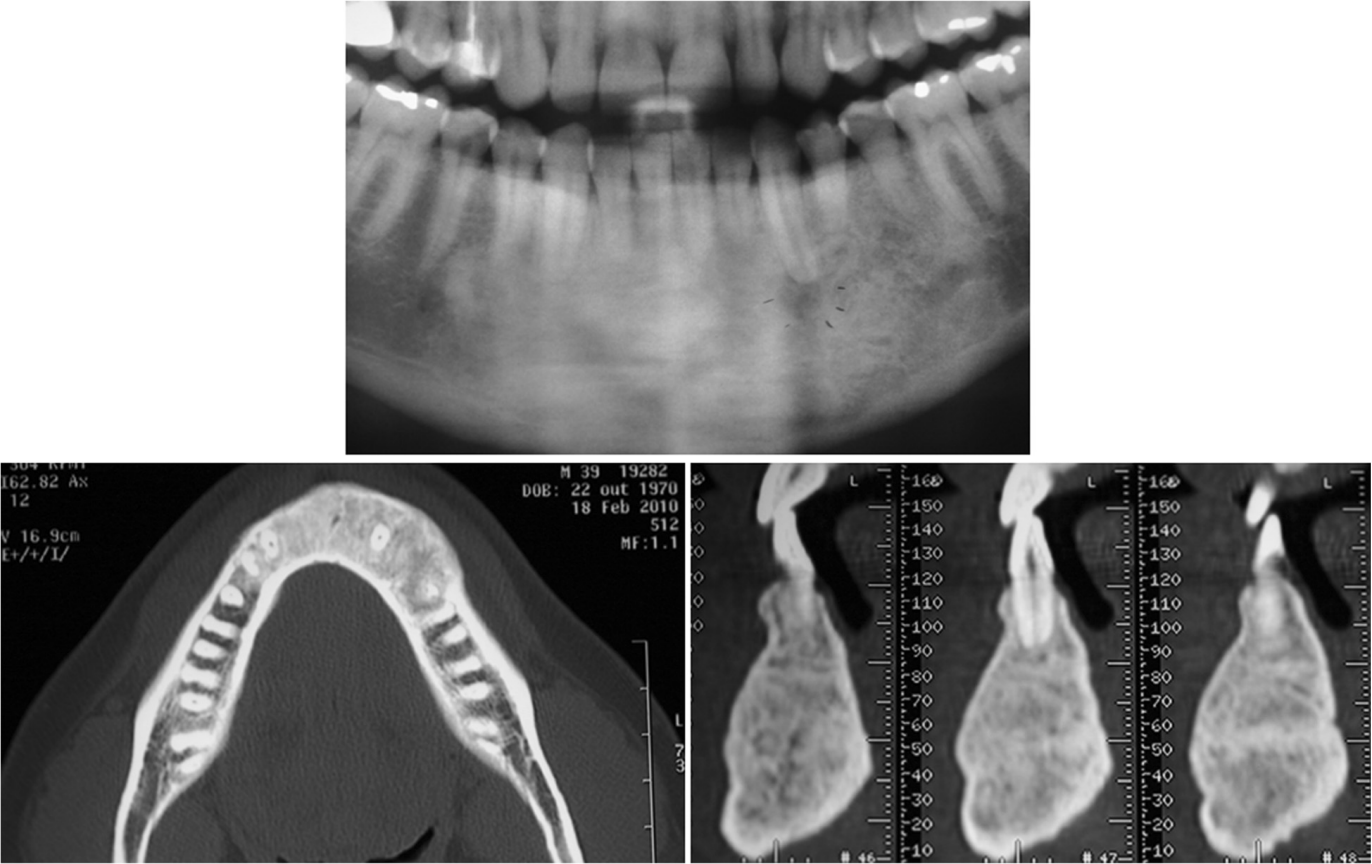
**Follow-up after two years.** The bony architecture was near normal, and only minimal radiolucency was present around the root apices of the involved teeth.

**Figure 5:**
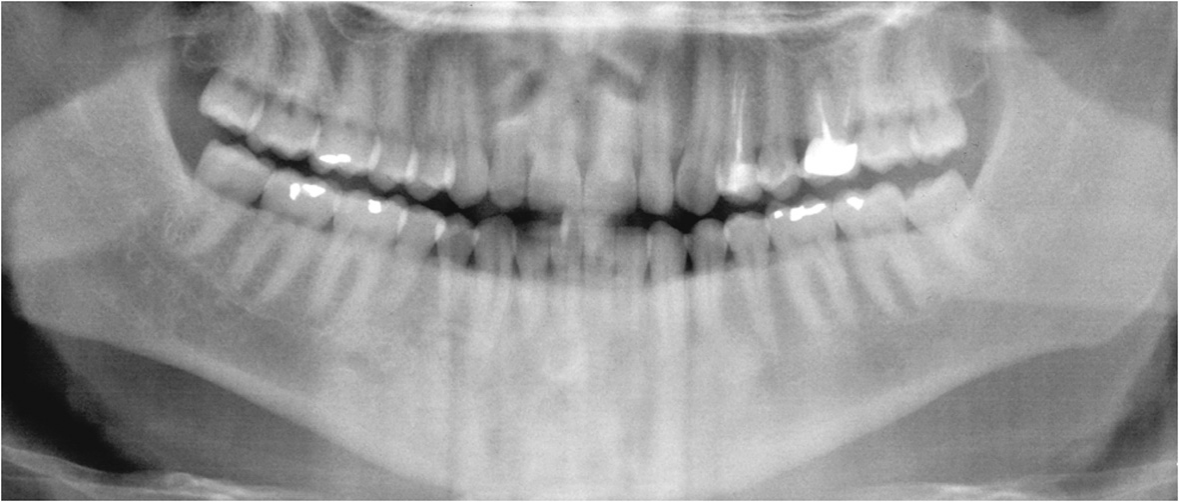
**Follow-up after four years.** Areas of new bone formation with more intense radiopacity.

After two years, the bony architecture was near normal and only minimal radiolucency was present around the root apices of the involved teeth. Both the cortical and the drilling areas proved to be repaired (Figure 4). The patient has been followed up for four years, and he is asymptomatic. The panoramic radiography and CT scan showed areas of bone formation with more intense radiopacity and no recurrence thus far (Figure 5).

## Discussion

CGCLs account for 10% of all benign jaw lesions. The proportion of mandibular to maxillary involvement is 2:1 or 3:1 [13]. Lesions are more common in the anterior region of the jaw, and mandibular lesions frequently extend across the midline [14], as observed in our case.

Based on clinical and radiographic features, these lesions can be classified as non-aggressive and aggressive. The non-aggressive form is characterised by slow growth, typically asymptomatic growth that does not perforate the cortical bone or induce root resorption and has a low recurrence rate. Aggressive lesions are characterised by pain, rapid growth, paraesthesia, root resorption, cortical perforation and a high recurrence rate after surgical treatment [5]. Although the patient did not report pain, other features of an aggressive lesion were observed, including paraesthesia, cortical perforation and root resorption.

A number of alternative nonsurgical therapies, such as intralesional corticosteroid injection, have been described for the management of CGCL. On reviewing the literature, we have found 18 reported cases that were treated with corticosteroids, with variable responses (Table 1). In the majority of the cases, six injections were administered, and the patients were followed up with periodic radiographic examination.

**Table 1:** Reported cases managed by intralesional corticosteroids injection

**Year**	**Author**	**No. of cases**	**Site**	**No. of injections**	**Time of resolution**
1994	Terry and Jacoway [12]	4	Mandible	6	3 years
			Mandible	6	1 year 4 months
			Mandible	6	2 years 2 months
			Mandible	6	Incomplete
1994	Kermer et al. [20]	1	Mandible	6	3 years
2000	Khafif et al. [21]	1	Maxilla	6	2 years
2001	Kurtz et al.[6]	1	Mandible	12	2 years
2001	Adornato and Paticoff [7]	1	Mandible	6	Partial 7 months
2002	Carlos and Sedano [8]	4	Maxilla	20	7 years
			Mandible	17	6 years
			Maxilla	4	Residual lesion 1 year 3 months
			Mandible	4	2 years
2005	Sezer et al. [9]	1	Mandible	6	3 years
2005	Abdo et al. [18]	1	Mandible	3	1 ½ year
2009	Mohanty and Jhamb [10]	2	Mandible	5	1 ½ year
			Mandible	6	1 ½ year
2010	Shirani et al. [19]	1	Mandible and maxilla	6	2 years
2011	Rachmiel et al. [11]	1	Mandible	6	5 years
Total	11	18	Proportion mandible:maxilla 15:4	Mean 7.27	Mean 2 ½ years

Steroid therapy was chosen in this case report because of the following advantages: ease of administration and lower invasiveness; relatively short duration of treatment (6 weeks average compared with 3–27 months for calcitonin and interferon-α); a relatively higher success rate compared with calcitonin/interferon-α; cost-effectiveness; availability; and minimal systemic side effects [10]. In addition, intralesional injection is preferable to systemic administration to achieve an elevated concentration of the medication in the tissue [7].

CGCL treatment is described as surgery, but the evidence of osteoclastic features enabled a change in the treatment of more aggressive cases, which tend to recur, or the cases with extensive destruction that require reconstructive surgery. In this case, the patient did not undergo surgery because he wished to preserve the nearby teeth and their vitality, to avoid endodontic treatments, and to preserve the maintenance of periodontal support and the structure of the adjacent bone.

It is well known that corticosteroids decrease blood calcium levels by suppressing intestinal calcium absorption and decreasing vitamin D activity and the reabsorption of calcium in the renal tubules [15]. In the present case, calcium carbonate was administered to facilitate the calcification of the lesion.

Bisphosphonates inhibit the formation of osteoclasts from immature precursor cells and induce the apoptosis of mature osteoclasts. Landesberg et al. [1] reported three cases of CGCLs treated with bisphosphonates with variable responses. It is evident in the two cases that the most significant regression is observed after the initial infusion of the drug. In the patient who received the drug at yearly intervals, it is difficult to determine whether higher doses and/or frequency of drug administration would have shown a more favourable result. The authors suggested that this drug might be useful as a primary or adjunctive therapy in the treatment of CGCL.

The administration of bisphosphonates in Paget’s disease, bone metastases of multiple myeloma, breast and prostate cancers and osteoporosis effectively restores bone mineral density and bone strength, reduces the incidence of bone fracture and dramatically improves quality of life [16].

Although bisphosphonates are generally well tolerated, bisphosphonate exposure has been linked to osteonecrosis of the jaw (ONJ). The most important predisposing factors for this condition are the type and total dose of bisphosphonate and a history of trauma, dental surgery or dental infection. The risk is substantially higher for patients taking zoledronic acid and increases over time, likely because of the long half-life of these drugs. The degree of risk for ONJ in patients taking oral bisphosphonates, such as alendronate, for osteoporosis is uncertain and warrants careful monitoring [17].

The combination of steroids with bisphosphonates appears to be valid because the former have been widely used in the treatment of CGCL [4,9-11,18-20], whereas the latter have been used successfully in antiresorptive bone lesions, such as osteoporosis and Paget’s diseases [1]. Although bisphosphonates have been successfully used to control bone resorption in various diseases [1,16].

Various possibilities of treatment or drug control increased the challenge of elucidating biomolecular characteristics at the time of diagnosis. We must not forget the possibility of changes that may occur with these developments, which emphasise the importance of biomolecular analysis [5]. It is also important to remember that these treatments until then described the main targets for the control of osteoclastogenesis, which is likely the expression of a genetic mutation or a metabolic disturbance. Therefore, research should continue to identify the factor that stimulates this local osteoclastogenesis and determine the most effective treatment.

## Conclusion

The treatment for CGCLs remains controversial because recurrence is possible. Although the surgical procedures are effective, they have poor aesthetic and functional results when large resections are performed. Unfortunately, in this study, the combination of alendronate with corticosteroids does not appear to have benefits in treating CGCL; however, only clinical trials with a large sample size will confirm the advantages of this association.

### Consent

Written informed consent was obtained from the patient for the publication of this case report and any accompanying images. A copy of the written consent is available for review by the Editor-in-Chief of this journal.

## Abbreviations

CGCL: Central giant cell lesion; CT: Computed tomography; ONJ: Osteonecrosis of the jaws.

## Competing interests

The authors declare that they have no competing interests.

## Authors’ contributions

NGSJ conceived of the study and participated in its design. ASDC drafted the manuscript. ENP and FMT participated in the analysis and interpretation of data. KLO assisted with the draft and review of the manuscript. JJVP participated in the design of the study and the coordination of the draft of the manuscript. All of the authors read and approved the final manuscript.
